# Injuries of neural tracts in a patient with CADASIL: a diffusion tensor imaging study

**DOI:** 10.1186/s12883-015-0434-x

**Published:** 2015-09-28

**Authors:** Sung Ho Jang, You Sung Seo

**Affiliations:** Department of Physical Medicine and Rehabilitation, College of Medicine, Yeungnam University, 317-1, Daemyungdong, Namku, Taegu, 705-717 Republic of Korea

**Keywords:** CADASIL, Diffusion tensor imaging, Diffusion tensor tractography

## Abstract

**Background:**

We report a patient with cerebral autosomal dominant arteriopathy with subcortical infarcts and leukoencephalopathy (CADASIL), who showed injuries of the neural tracts, which was demonstrated by diffusion tensor tractography (DTT).

**Case presentation:**

A 64-year-old male patient and seven age-matched control volunteers were recruited. Since approximately 1.5 years ago, he had felt mild weakness of the right arm and was diagnosed as CADASIL by the finding of the exon 11 mutation of the NOTCH3 gene approximately 10 months ago. T2-weighted and FLAIR brain MRI images obtained at admission showed high signal intensity lesions in the subcortical gray matter and periventricular white matter. He showed mild quadriparesis, mild dysarthria, mild cognitive impairment, and emotional problems. Diffusion tensor imaging was performed and nine neural tracts (corticospinal tract, corticobulbar tract, corticofugal tract from the supplementary motor area, corticofugal tract from the premotor cortex, thalmoprefrontal tract [TPT] to the dorsolateral prefrontal cortex, TPT to the ventrolateral prefrontal cortex, TPT to the orbitoprefrontal cortex, fornix, and cingulum) were reconstructed. Fractional anisotropy (FA), mean diffusivity (MD), and tract volume of each neural tract were measured. All neural tracts except for the left fornix showed at least one more abnormality in terms of DTT parameters (decrement of FA, increment of MD, or decrement of tract volume).

**Conclusion:**

We demonstrated injuries of the neural tracts in a patient with CADASIL. It appears that clinical manifestations in this patient were related to injuries of the neural tracts.

## Background

Cerebral autosomal dominant arteriopathy with subcortical infarcts and leukoencephalopathy (CADASIL) is an inherited small artery disease caused by mutations in the NOTCH3 gene [1–3]. The clinical manifestation of CADASIL is known to be variable, including five main symptoms: migraine with aura, subcortical ischemic events, mood disturbances, apathy, and cognitive impairment [3]. This suggests that various neural tracts in the human brain might be vulnerable to CADASIL, however, little is known about this, even though elucidation of injuries of neural tracts is important in terms of diagnosis and rehabilitation. Conventional brain MRI is known to be sensitive in detecting lesions of CADASIL, therefore, it has been considered as an important diagnostic tool of CADASIL although it has significant limitation in precise evaluation of the neural tracts. By contrast, diffusion tensor tractography (DTT), which is derived from diffusion tensor imaging (DTI), has enabled three-dimensional visualization and estimation of the neural tracts. Since the introduction of DTI, several DTI studies have reported on the usefulness of DTI for detection of lesions of CADASIL [4–8]. However, no study on neural tract injury in patients with CADASIL has been reported so far.

In the current study, we report on a patient with CADASIL, who showed injuries of the neural tracts, which was demonstrated by DTT.

## Case Presentation

### Subjects and methods

#### Subjects

A 64-year-old, right-handed male patient was admitted for rehabilitation at the department of rehabilitation in a university hospital. Approximately 1.5 years ago, he suddenly felt mild weakness of the right arm, consequently, he had mild difficulty in writing and using chopsticks. At a local neurology clinic, he was diagnosed with multiple lacunar infarct and was prescribed aspirin. Approximately 10 months ago, he began to show gait disturbance and mild dysarthria. At the neurology department of another university hospital, he was as diagnosed as CADASIL by the finding of the exon 11 mutation of the NOTCH3 gene. With the passage of time, he began to show irritability, depression, and memory impairment. However, he had no history of migraine, and his father had suffered from stroke which was not accurately diagnosed.

**Fig. 1:**
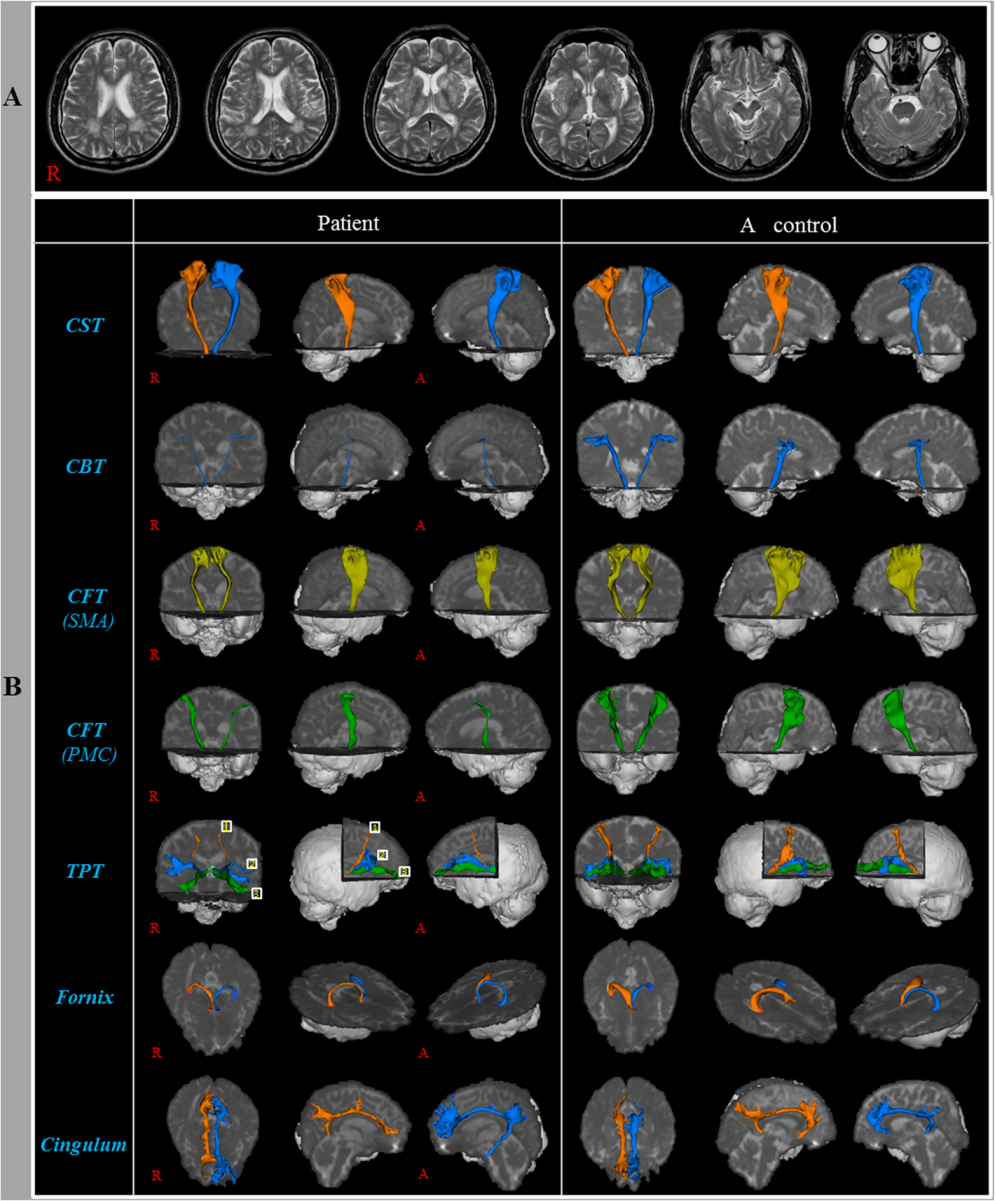
**a** T2-weighted brain MR images obtained at admission show high signal intensity lesions in the subcortical gray matter and periventricular white matter. **b** Diffusion tensor tractographies for the patient and a control volunteer man (58-year old male). CST: Corticospinal tract, CBT: Corticobulbar tract, CFT: Corticofugal tract, SMA: Supplementary motor area, PMC: Premotor area, TPT: Thalamoprefrontal tract; 1: Dorsolateral prefrontal cortex, 2: Ventrolateral prefrontal cortex, 3: Orbito prefrontal cortex

Brain MRI (T2-weighted and Fluid attenuated inversion recovery [FLAIR] images) performed at admission showed high signal intensity lesions in the subcortical gray matter and periventricular white matter (Fig. 1-a). On the neurological examination, he showed mild quadriparesis (4^+^/4^+^) with limb-kinetic apraxic movement pattern of the right extremities (slow and clumbsy), mild dysarthria, and mild aspiration sign (reflexive cough when drinking water). He also showed mild cognitive impairment and emotional problems as follows: Mini-Mental State Examination (28), Wechsler Adult Intelligence Scale (97), Memory Assessment Scale; global memory (94: 35%ile), Clinical Dementia Rating scale (1.5), Modified Neuropsychiatric Inventory: irritability (6: 0 ~ 12), depression (6: 0 ~ 12), disinhibition (6: 0 ~ 12), and the ideomotor apraxia test (40: cut-off score < 32).

Seven age-matched control volunteers (three male; mean age: 55.7 years, range: 52–62) with no history of neurologic disease participated in this study. All volunteers provided signed, informed consent, and the study protocol was approved by our institutional review board.

### Diffusion tensor imaging

DTI data were acquired at admission (approximately 1.5 years after onset of initial symptoms) using a 6-channel head coil on a 1.5 T Philips Gyroscan Intera (Philips, Best, Netherlands) with single-shot echo-planar imaging. For each of the 32 non-collinear diffusion sensitizing gradients, we acquired 67 contiguous slices parallel to the anterior commissure-posterior commissure line. Imaging parameters were as follows: acquisition matrix = 96 × 96, reconstructed to matrix = 128 × 128, field of view = 221 × 221 mm^2^, TR = 10,726 ms, TE = 76 ms, parallel imaging reduction factor (SENSE factor) = 2, EPI factor = 49 and b = 1000 s/mm^2^, NEX = 1, and a slice thickness of 2.3 mm (acquired voxel size 1.73 × 1.73 × 2.3 mm^3^).

### Probabilistic fiber tracking

Analysis of diffusion-weighted imaging data was performed using the Oxford Centre for Functional Magnetic Resonance Imaging of the Brain (FMRIB) Software Library (FSL; www.fmrib.ox.ac.uk/fsl). Affine multi-scale two-dimensional registration was used for correction of head motion effect and image distortion due to eddy current. Fiber tracking was performed using a probabilistic tractography method based on a multifiber model, and applied in the current study utilizing tractography routines implemented in FMRIB Diffusion (5000 streamline samples, 0.5 mm step lengths, curvature thresholds = 0.2).

For analysis of the nine neural tracts, a seed and target regions of interest (ROIs) were placed as follows and each tract was constructed by selection of fibers passing through two ROIs: corticospinal tract (CST): anterior portion of the pons, primary motor cortex; corticobulbar tract (CBT): lower portion of the precentral gyrus, lower pons (anterior blue portion); corticofugal tract (CFT) from the supplementary motor area (SMA): supplementary motor area, cerebral peduncle of midbrain; CFT from the premotor cortex (PMC): premotor cortex area, cerebral peduncle of midbrain; thalmoprefrontal tract (TPT) to the dorsolateral prefrontal cortex (DLPFC): mediodorsal nucleus of the thalamus, Brodmann areas (BAs) (8, 9, and 46); TPT to the ventrolateral prefrontal cortex (VLPFC): mediodorsal nucleus of the thalamus, BAs (44, 45, and 47); TPT to the orbitoprefrontal cortex (OFC): MD, BAs (47/12, 10, 11, and 13); fornix: mammillary body, crus of the fornix; cingulum: middle portion of the cingulum, posterior portion of the cingulum. Of 5000 samples generated from the seed voxel, contact results were visualized at a threshold minimum of 1 streamline through each voxel for analysis. Fractional anisotropy (FA), mean diffusivity (MD), and tract volume in nine neural tracts (CST, CBT, CFT from the SMA, CFT from the PMC, TPT to the DLPFC, TPT to the VLPFC, TPT to the OFC, fornix and cingulum) were measured.

A summary of the DTT parameters of the nine neural tracts in the patient and normal control volunteers is shown in Table 1. DTT parameter values showing a deviation of more than two standard deviations from normal control values were defined as abnormal. All neural tracts except for the left fornix showed at least one or more abnormality in terms of DTT parameters (the decrement of FA, and/or the increment of MD, and/or the decrement of tract volume) and the abnormalities of DTT parameters were as follows: FA value was decreased on both sides of the CST, the CFT from the SMA, the TPT to the OFC, the cingulum, the left side of the TPT to the DLPFC, the right side of the TPT to the VLPFC and the fornix. MD value was increased on both sides of the CBT, the TPT to the DLPFC, the TPT to the VLPFC, the TPT to the OFC and the cingulum, and the right side of the CST and the fornix. Tract volume was decreased on both sides of the CBT, the CFF from the PMC, the TPT to the DLPFC, the TPT to the OFC, left side of the TPT to the VLPFC.

**Table 1:** Diffusion tensor image parameter values of patient and control volunteers

Neural tracts		Patient			Controls		
		FA	MD	Tract volume	FA	MD	Tract volume
CST	Rt	0.34^a^	1.06^b^	3705	0.40 (0.01)	0.87 (0.04)	4099.00 (317.00)
	Lt	0.32^a^	1.01	4386	0.41 (0.02)	0.90 (0.09)	3993.00 (549.00)
CBT	Rt	0.49	1.41^b^	183^a^	0.47 (0.03)	0.80 (0.03)	1030.14 (385.98)
	Lt	0.48	1.13^b^	306^a^	0.48 (0.02)	0.81 (0.05)	1310.29 (307.93)
CFT (SMA)	Rt	0.27^a^	1.15	5097	0.37 (0.02)	0.99 (0.09)	6322.00 (746.02)
	Lt	0.28^a^	1.10	5209	0.38 (0.02)	0.94 (0.08)	6205.71 (1229.92)
CFT (PMC)	Rt	0.39	0.86	893^a^	0.37 (0.02)	0.88 (0.05)	2412.14 (706.68)
	Lt	0.40	0.94	229^a^	0.39 (0.03)	0.87 (0.06)	2524.57 (819.62)
TPT (DLPFC)	Rt	0.32	0.99^b^	83^a^	0.36 (0.02)	0.81 (0.04)	592.00 (156.03)
	Lt	0.30^a^	1.00^b^	133^a^	0.36 (0.02)	0.82 (0.05)	590.14 (221.59)
TPT (VLPFC)	Rt	0.28^a^	1.01^b^	117	0.37 (0.02)	0.82 (0.07)	772.86 (367.67)
	Lt	0.37	0.99^b^	286^a^	0.37 (0.02)	0.82 (0.03)	873.86 (224.90)
TPT (OFC)	Rt	0.27^a^	1.00^b^	347^a^	0.34 (0.04)	0.81 (0.06)	1439.71 (432.18)
	Lt	0.27^a^	1.01^b^	817^a^	0.36 (0.03)	0.82 (0.06)	1149.43 (164.44)
Fornix	Rt	0.27^a^	1.64^b^	418	0.34 (0.02)	1.41 (0.05)	560.20 (151.00)
	Lt	0.28	1.57	448	0.34 (0.04)	1.49 (0.14)	421.00 (216.71)
Cingulum	Rt	0.23^a^	0.91^b^	2378	0.33 (0.02)	0.85 (0.02)	2177.50 (286.64)
	Lt	0.28^a^	0.98^b^	4824	0.338 (0.03)	0.88 (0.03)	2046.30 (284.34)

Controls are mean (± standard deviation), FA: fractional anisotropy, MD: mean diffusivity, CST: Corticospinal tract, CBT: Corticobulbar tract, CFT: Corticofugal tract, SMA: Supplementary motor area, PMC: Premotor area, TPT: Thalamoprefrontal tract, DLPFC: Dorsolateral prefrontal cortex, VLPFC: Ventrolateral prefrontal cortex, OFC: Orbitoprefrontal cortex

^a^when a value was decreased two standard deviations below that of controls

^b^when a value was increased two standard deviations above that of controls

## Conclusions

In the current study, we investigated injury of the neural tracts (CST, CBT, CFT [SMA], CFT [PMC], TPT [DLPFC], TPT [VLPFC], TPT [OFC], fornix, cingulum), which are mainly located in the frontal lobe in a patient with CADASIL. We found that all neural tracts, except for the left fornix, which we estimated showed at least one more abnormality in terms of DTT parameters (the decrement of FA, and/or the increment of MD, and/or the decrement of tract volume). FA value represents the degree of directionality of microstructures (e.g., axons, myelin, and microtubules) and MD value indicates the magnitude of water diffusion. By contrast, tract volume is determined by the number of voxels contained within the neural tract. Therefore, the decrement of FA value or tract volume, or the increment of MD value of a neural tract appeared to indicate a neural injury. Consequently, this patient’s clinical manifestations (quadriparesis, limb-kinetic apraxia, cognitive impairment, and emotional problems) appear to be related to injury of the above mentioned neural tracts.

In conclusion, we demonstrated injuries of the neural tracts in a patient with CADASIL. It appears that clinical manifestations in this patient were related to injuries of the neural tracts. Therefore, we suggest that evaluation of the neural tracts which are relevant with clinical manifestations would be useful in patients with CADASIL. To the best of our knowledge, this is the first DTT study demonstrating injury of the neural tracts in a patient with CADASIL, although several DTI studies have demonstrated injury of some regions of subcortical white and gray matter in patients with CADASIL. However, limitations of this study should be considered. First, DTT may underestimate or overestimate the fiber tracts because regions of fiber complexity and crossing can prevent full reflection of the underlying fiber architecture by DTT. Second, because it is a case report, this study is limited; therefore, conduct of further studies involving large number of patients and long-term follow up studies should be encouraged.

## Consent

Written informed consent was obtained from the patient for publication of this case report and accompanying images. A copy of the written consent is available for review by the Editor-in-Chief of this journal.

## Abbreviations

DTI: Diffusion tensor imaging
DTT: Diffusion tensor tractography
ROI: Region of interest
CST: Corticospinal tract
CBT: Corticobulbar tract
CFT: Corticofugal tract
SMA: Supplementary motor area
PMC: Premotor cortex
TPT: Thalamoprefrontal tract
DLPFC: Dorsolateral prefrontal cortex
BAs: Brodmann areas
VLPFC: Ventrolateral prefrontal cortex
OFC: Orbitoprefrontal cortex
FA: Fractional anisotropy
MD: Mean diffusivity
